# Alterations of tumor microenvironment by carbon monoxide impedes lung cancer growth

**DOI:** 10.18632/oncotarget.8081

**Published:** 2016-03-15

**Authors:** Zsuzsanna Nemeth, Eva Csizmadia, Lisa Vikstrom, Mailin Li, Kavita Bisht, Alborz Feizi, Sherrie Otterbein, Brian Zuckerbraun, Daniel B. Costa, Pier Paolo Pandolfi, Janos Fillinger, Balazs Döme, Leo E. Otterbein, Barbara Wegiel

**Affiliations:** ^1^Department of Surgery, Transplant Institute, Beth Israel Deaconess Medical Center, Harvard Medical School, Boston, MA, USA; ^2^Cancer Center Institute, Beth Israel Deaconess Medical Center, Harvard Medical School, Boston, MA, USA; ^3^Department of Medicine, Beth Israel Deaconess Medical Center, Harvard Medical School, Boston, MA, USA; ^4^Department of Tumor Biology, National Koranyi Institute of TB and Pulmonology, Budapest, Hungary; ^5^Heart Foundation Research Center, Griffith Health Institute, Griffith University, Gold Coast, Australia; ^6^Department of Surgery, Section of Trauma and Acute Care Surgery, University of Pittsburgh Medical Center, Pittsburgh, PA, USA; ^7^Department of Pathology, National Koranyi Institute of TB and Pulmonology, Budapest, Hungary; ^8^Division of Thoracic Surgery, Department of Surgery, Comprehensive Cancer Center, Medical University of Vienna, Austria; ^9^Department of Thoracic Surgery, National Institute of Oncology, Budapest, Hungary

**Keywords:** tumor microenvironment, carbon monoxide, macrophages, immunotherapy

## Abstract

We hypothesized that tumor-associated macrophages (TAMs) are controlled by the diffusible gas carbon monoxide (CO). We demonstrate that induction of apoptosis in lung tumors treated with low doses of CO is associated with increased CD86 expression and activation of mitogen-activated protein kinase (MAPK)/extracellular signal-regulated kinases (Erk) 1/2 pathway in tumor microenvironment. Presence of CD86-positive cells was required for the anti-tumoral effects of CO in established A549 xenografts. We show that the effects of CO on tumor stroma and reprogramming of macrophages towards the anti-tumoral phenotype is mediated by reactive oxygen species (ROS)-dependent activation of MAPK/Erk1/2-c-myc pathway as well as Notch 1-dependent negative feedback on the metabolic enzyme heme oxygenase-1 (HO-1). We find a similar negative correlation between HO-1 and active MAPK-Erk1/2 levels in human lung cancer specimens.

In summary, we describe novel non-cell autonomous mechanisms by which the diffusible gas CO dictates changes in the tumor microenvironment through the modulation of macrophages.

## INTRODUCTION

Heme catalysis by the heme oxygenases (HO) generates CO next to bilirubin and iron. CO has potent immunomodulatory functions and can influence innate immune cell recruitment as well as myeloid cell differentiation [1–3]. HO-1 is detected primarily in the nucleus of cancer cells in an altered enzymatically inactive form, generating limited amounts of CO [4]. We have recently shown that deletion of HO-1 in macrophages decreases the growth rate of prostate cancer, yet leads to higher metastatic outgrowth [5]. Overexpression of HO-1 in cancer cells blocks A549 lung carcinoma xenografts growth [5–7], likely through higher levels of CO.

We recently showed that CO at 250 ppm targeted mitochondrial respiration and glucose metabolism in cancer cells and thus reduced tumor growth [8]. Since CO targets multiple cell types, we reasoned that application of exogenous CO may have potent anti-tumoral activity not only directly on cancer cells, but also importantly on immune cells in the tumor microenvironment. A role of exogenous CO in TAMs has not been tested previously and was a focus of this study. Based on previous work on CO effects on macrophages [9, 10], we asked whether exogenous application of CO could influence macrophage polarization in the tumor microenvironment and what effect this would have on cancer initiation and progression. TAMs are polarized towards a pro-angiogenic, IL-10 producing M2 phenotype and are associated with poor prognosis [11, 12]. In contrast, pro-inflammatory M1 classically activated macrophages, expressing high levels of CD86 and CD80, reinforce anti-tumoral immune responses [13–15]. Macrophages expressing high levels of HO-1 have been associated with poor outcome of cancer patients [16]. In contrast, lower expression of HO-1 in TAMs was detected in Non-Small Cell Lung Cancer (NSCLC) as compared to in tumor-free lung residential macrophages [17]. In this study, we asked whether CO affects cancer growth via skewing of macrophages towards the classically activated M1-like phenotype.

In this study, we demonstrate the importance of CO in the tumor microenvironment. By modulating HO-1 expression, CO affects the phenotype and polarization status of macrophages in lung cancer. Further, we show that CD86-postive macrophages are required for anti-tumoral effects of CO. ROS-driven Erk1/2→Notch1 signaling pathway and blockage of HO-1 expression are the prototypic characteristics of macrophages in the tumor microenvironment upon CO treatment. In summary, we show that a balance of HO-1 and CD86 expressions in myeloid cells in response to CO treatment is critical for host responses during progression of lung cancer.

## RESULTS

### Exogenous CO at low doses blocks progression of lung cancer which correlates with increased Erk1/2-c-myc activity

**Figure 1:**
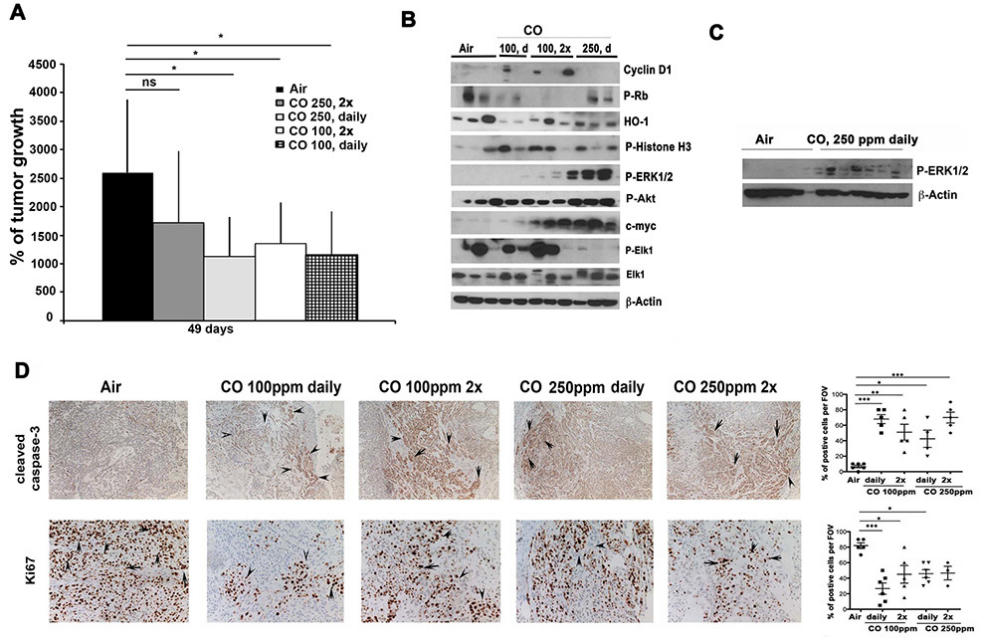
Low doses of CO block growth of lung cancer xenografts **A–B.** Tumor volumes of A549 xenografts established in nude mice for 2 weeks and then treated with air, CO (1h, 250 ppm) twice per week (2x), CO (1h, 250 ppm) daily or CO (1h, 100 ppm) twice per week (2x), CO (1h, 100 ppm) daily for 49 days. % of tumor growth at 49 days versus the time zero when CO treatments started are shown. n=4-8 tumors per group. Averages±SD. * p<0.05: CO (different doses) treated versus air at day 49. Analyses of all treatment time points: p<0.05 for CO versus air for all treatments at day 45 and 49. p<0.05 for CO 2x week at 100 ppm at 37 days. **B.** Protein lysates of xenograft tumor samples from A were analyzed by immunoblotting for expression of cell cycle proteins and activity of signaling pathways. **C.** Lewis lung carcinoma (CRL) syngeneic model in C57BL/6 mice were established for a week and treated with 250 ppm CO daily for 1h for a week. Immunoblotting of whole lysates of tumors was performed testing expression of P-Erk1/2. n=6 (Air), n=8 (CO) tumors per group (n=3-4 mice per group). **D.** Immunohistochemistry of A549 xenograft tumors was performed with antibodies for detection of apoptosis (Cleaved caspase-3) and proliferation (Ki67).

To understand the mechanism of diffusible CO gas in tumor stroma and to gain insight into potential clinical application of CO as an adjuvant for cancer treatment, we investigated the effect of two different CO regimens on lung cancer growth. Mice with established A549 lung carcinoma xenografts were exposed to CO daily or twice per week at 100 ppm or 250 ppm for 1 hour. Surprisingly, we found that treatment with CO at 100 ppm daily and 100 ppm twice per week were equally effective as 250 ppm daily for 1 hour in blocking tumor growth (Figure 1A). Since lower doses of CO were equally effective as higher doses of CO, we asked the question whether both work through the same mechanism. No significant changes in total expressions of cell cycle regulatory proteins cyclin D1, phosphorylated retinoblastoma protein (Rb) or HO-1 and phosphorylated Histone H3 were noted in response to treatment with different doses of CO (Figure 1B, Supplementary Figure S1A–S1D). Further, there was no difference in phosphorylation of MAPK p38 or MAPK JNK in response to CO in tumor xenografts (data not shown). In contrast, we observed significant induction of phosphorylated MAPK-Erk1/2 (Erk1/2) and Akt in mice treated with CO at 250 ppm daily (Figure 1B-1C, Supplementary Figure S1E-S1F). C-myc, a downstream target of the Erk1/2 pathway, was also strongly elevated in A549 xenograft tumors in mice treated with CO at 100 ppm twice per week and 250 ppm daily (Figure 1B, Supplementary Figure S1G) and unexpectedly correlated with strong suppression of tumor growth (Figure 1A). Further, activation of Erk1/2-c-myc correlated with slight decrease in P-Elk1 (Ets-like gene 1) in mice treated with CO at 250 ppm daily (Figure 1B, Supplementary Figure S1H). These findings were further confirmed in Lewis lung carcinoma CRL syngeneic model in C57BL/6 mice where application of CO for 7 days at 250 ppm daily for 1 hour in mice with established tumors led to increased phosphorylation of Erk1/2 (Figure 1C) and lower staining of proliferation marker Ki67 (data not shown). Suppression of tumor growth by CO in A549 xenografts corresponded to induction of caspase-3 cleavage in cancer cells indicating increased apoptosis (Figure 1D). Caspase-3 activation was accompanied by significantly decreased Ki67 staining in A549 xenografts from mice treated with CO (Figure 1D).

### CO modulates myeloid cell/macrophage infiltration and phenotype in the tumor microenvironment

**Figure 2:**
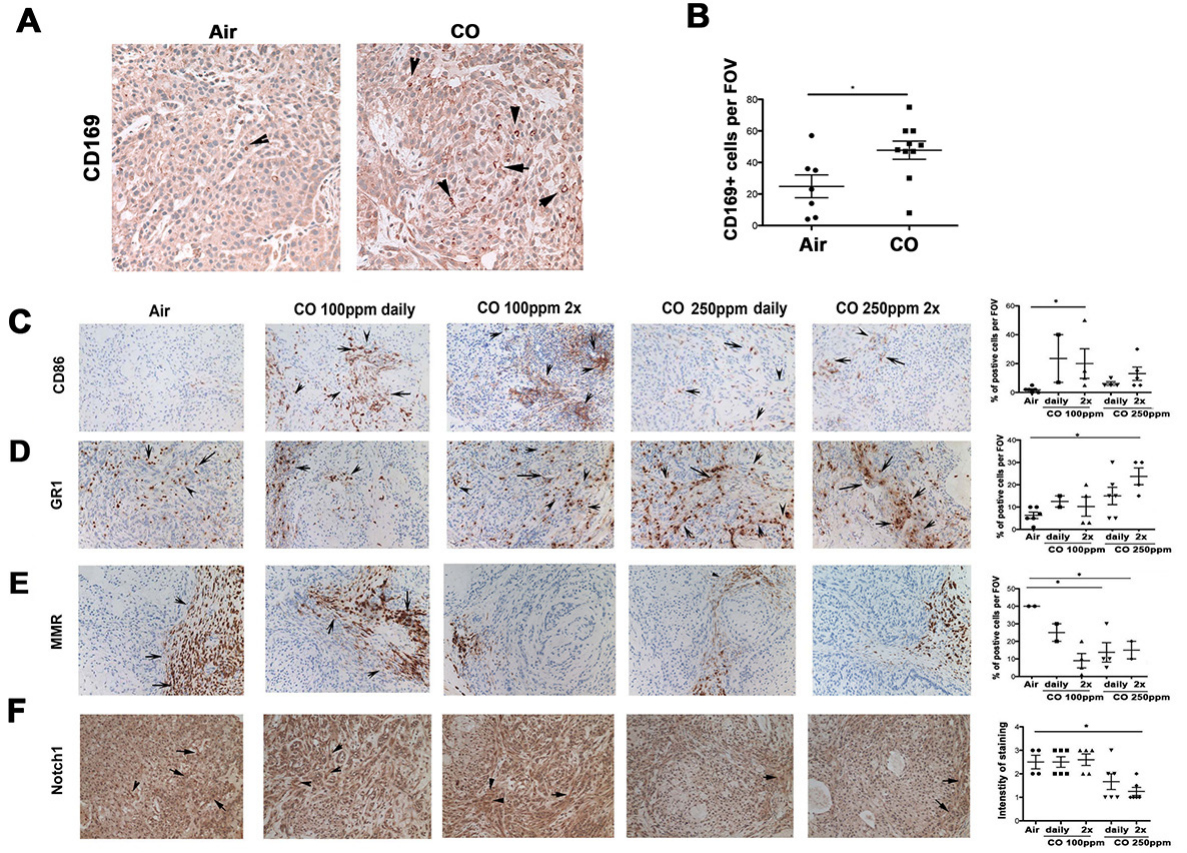
CO enhances infiltration of myeloid cells into tumor microenvironment or their skewing towards M1-like macrophages and additionally increases apoptosis in tumor cells **A–F.** Immunohistochemistry of A549 xenograft tumors as in Figure 1 (A549 xenografts established in nude mice and treated with 100 ppm or 250 ppm CO (1h) given daily or twice per week (2x)) was performed with antibodies for detection of myeloid cell markers. Quantification of n=2-6 sections is shown. **A–B.** Immunohistochemical analysis of CD169, phagocytic macrophage marker. **C.** CD86, mature M1-like macrophage and antigen presenting cell (APC) marker. **D.** Gr-1, granulocytes marker. **E.** MMR (CD206), M2-like macrophage marker **F.** Total Notch 1, for detection of the receptor expression level of activation signaling pathway in M1-like macrophages. *p<0.05, **p<0.01, *** p<0.001 CO treated versus air control. Magnification 200x. Quantification was performed by evaluating % of positive area per field of view. Arrows indicate cells positive for staining.

Since our studies suggest that CO has anti-proliferative and pro-apoptotic effects in tumors *in vivo* but only moderate direct effects on cancer cell growth *in vitro* [8, 18, 19] (Supplementary Figure S2A), we reasoned that the effects on tumor suppression were likely due to additional mechanisms by which CO influenced the tumor microenvironment. Indeed, we observed that CO treatment at 100 ppm led to increased number of CD169+ macrophages in the tumor microenvironment (Figure 2A-2B), which were previously shown to have strong anti-tumoral effects due to enhanced phagocytosis of dead tumor cells [20]. We have previously shown that CO increases phagocytic activity of macrophages in a model of bacteria infection [10]. Interestingly, CO at 100 ppm induced expression of M1 macrophage marker CD86, indicating skewing of this specific population in the tumor microenvironment (Figure 2C). In contrast, treatment with 250 ppm resulted in higher numbers of Gr-1^+^/CD11b^−^ myeloid cells (Figure 2D, data not shown), but the differential recruitment or maturation of myeloid cells was associated with similar outcomes as measured by tumor growth (Figure 1). Number of MMR-positive myeloid cells (M2 skewed) was significantly suppressed after CO treatment at each dose, suggesting a switch towards the M1 phenotype (Figure 2E). Decreased Notch1 total staining in tumor xenografts after treatment with higher doses of CO suggests cleavage and activation of Notch1 signaling which is characteristic of M1 macrophage skewing (Figure 2F).

### CO targets Notch1 and Erk1/2 signaling in vivo in tumor stroma – role of CD86+ myeloid cells

**Figure 3:**
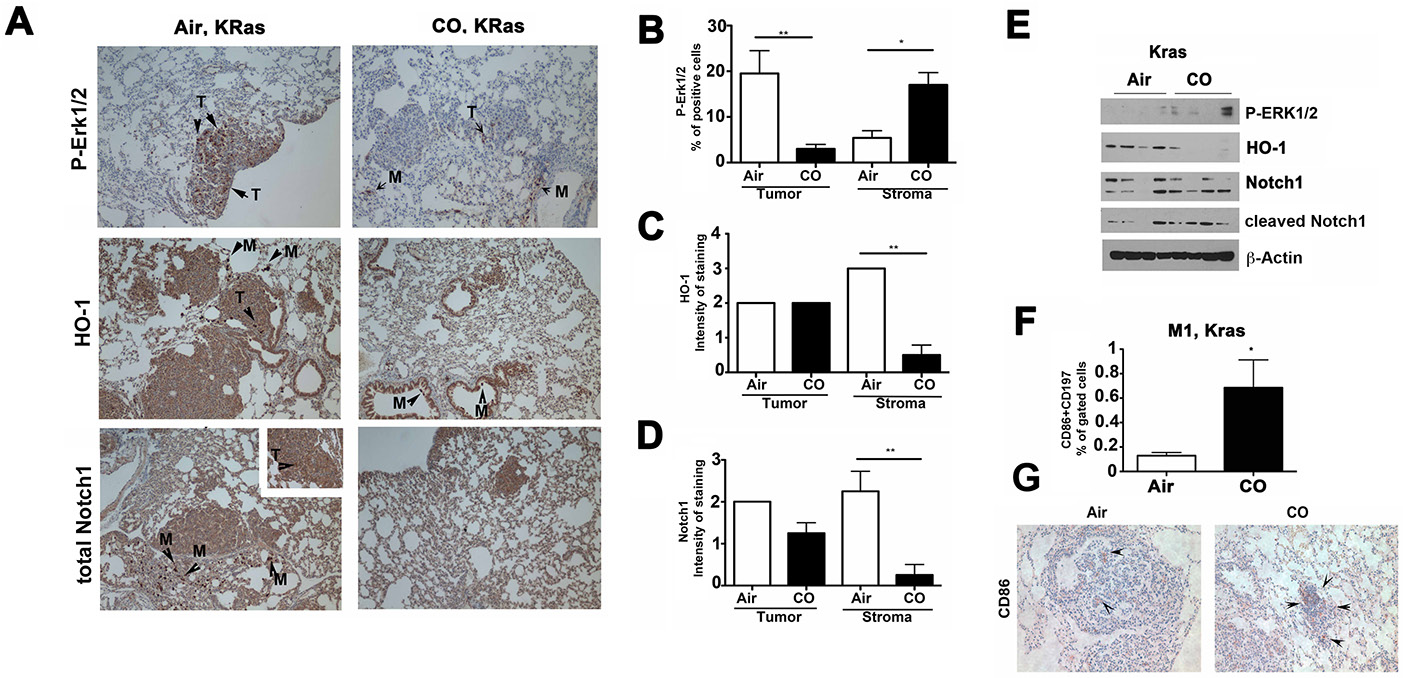
CO activates Erk1/2 and Notch1 signaling in Kras model of lung cancer **A.** Immunohistochemistry with antibodies against P-Erk1/2, HO-1 and total Notch1 in Kras tumors from mice treated with Air or CO (250ppm) for 5 weeks daily after initial establishment of tumors for 13 weeks. **B–D.** Semiquantitative evaluation of stainings, n=4-5 mice per group. Evaluation of number of cells or % of positive area per field of view at 200x in the stroma or in the lung cancer nodules was performed. **E.** Western blot analysis of Kras tumors treated as in A. n=4/group. **F.** Flow cytometry analysis of Kras tumor samples for expression of CD86 and CD197, markers of M1-like phenotype of macrophages. n=3/group. *p<0.05 CO versus Air. **G.** Immunohistochemistry with antibody against CD86 was performed on tissues of Kras tumors treated as in A. n=3/group. Arrows indicate CD86-positive cells in the tumor area.

To better understand the role of CO in modulating tumor microenvironment, we employed the Kras-driven spontaneous lung carcinoma model [8]. We show decreased numbers of nodules and lower Erk1/2 phosphorylation (P-Erk1/2) in tumor cells but increased P-Erk1/2 staining in the stroma cells upon treatment with CO (Figure 3A, 3B, 3E; Supplementary Figure S3A). Importantly, phosphorylation of Erk1/2 *in vitro* upon CO treatment was dependent on mitochondria generated ROS [8] as pre-treatment of RAW macrophages with pegylated-superoxide dismutase and pegylated-catalase blocked CO-induced P-Erk1/2 (Supplementary Figure S2B). We also showed that CO significantly enhanced cleavage of Notch1 (Figure 3E, Supplementary Figure 3C), which corresponded to decreased Notch1 expression in the stroma surrounding lung cancers in Kras mice treated with CO (Figure 3A, 3D). Interestingly, CO blocked expression of HO-1 in the stroma, suggesting a negative loop of heme degradation pathway in tumor microenvironment (Figure 3A, 3C, 3E). HO-1 is associated with M2 polarization of myeloid cells [21]. To evaluate whether CO modulates polarization of myeloid cells in tumor stroma in this model, we measured the number of CD86/CD197-postive M1-like myeloid cells by flow cytometry. We observed a significant increase in CD86^high^/CD197^high^ cells in lung stroma after treatment with CO (Figure 3F). Further, we saw higher amount of CD86 positive cells in the regions of regressing tumors in CO treated mice (Figure 3G), in line with CO treated A549 xenografts (Figure 2C).

**Figure 4:**
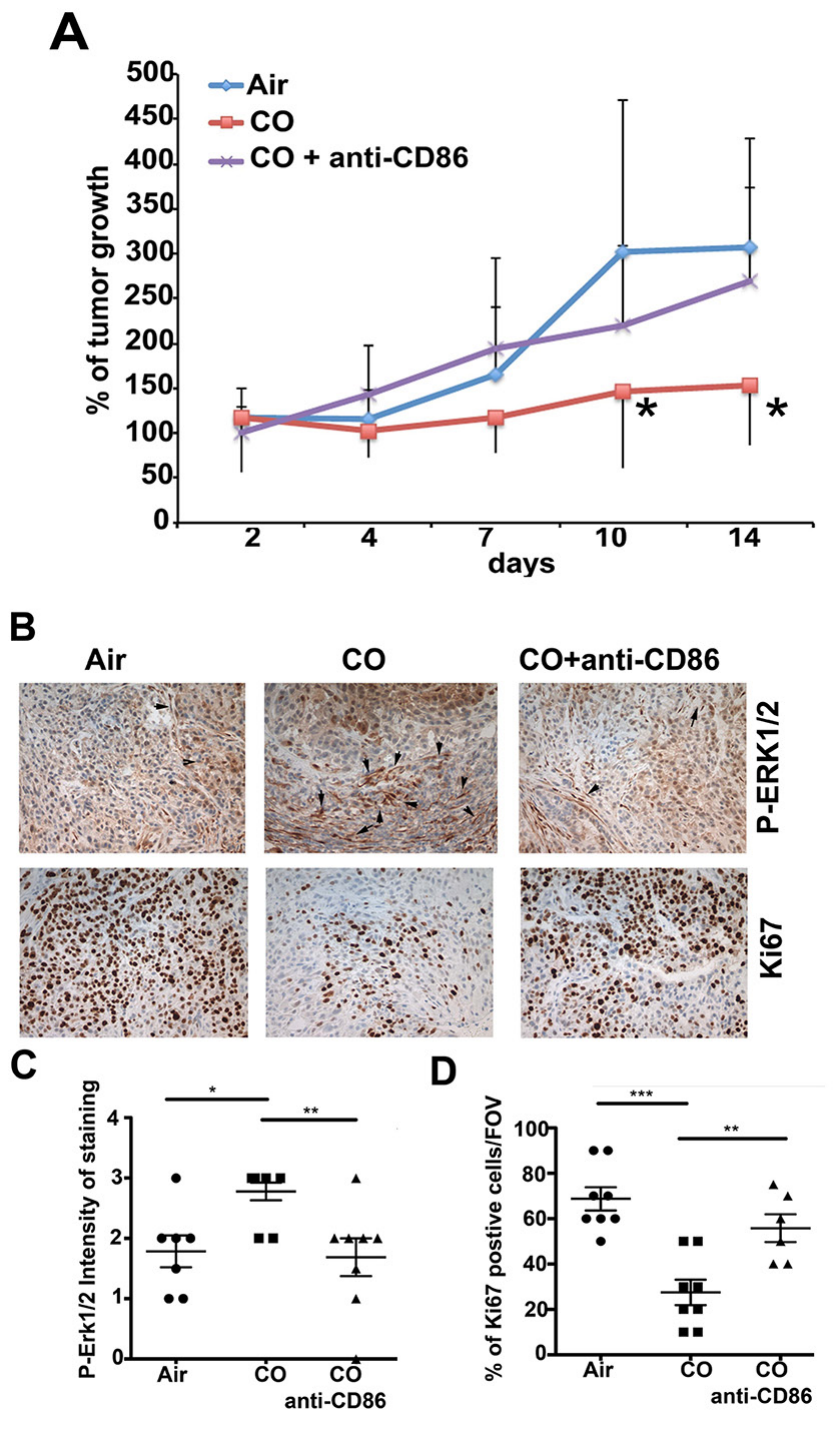
Requirement of CD86^high^ cells in tumor niche for CO effects **A.** Growth curves of A549 xenografts established in nude mice for 2 weeks and treated with 100 ppm CO (1h) twice per week ±CO ± anti-CD86 neutralizing antibody (100 mg/mouse, i.p.). % of tumor growth as compared to size at initiation of CO treatment as averages±SD. n=6-8 tumors per group. *p<0.05: CO versus Air. **B–D.** Immunohistochemistry with antibodies against P-Erk1/2 and Ki67 in xenograft tumors treated as in **A.** Quantification of staining is shown in C (P-Erk1/2) and D (Ki67) from n=4-5 mice per group. Evaluation is presented as intensity of staining (scored on a 1-4 scale with 1-low staining and 4-very strong staining) or % of positive area per field of view under 200x magnification. *p<0.05, **p<0.01, ***p<0.001.

To functionally test the role of CD86-positive myeloid cells in the tumor microenvironment in anti-tumoral effects of CO treatment, we depleted the CD86-postive population using anti-CD86 neutralizing antibody (Figure 4A). Inhibitory effects of CO on tumor growth were blocked with anti-CD86 neutralizing antibody, suggesting importance of CD86 positive population in mediating CO effects (Figure 4A). CO-mediated increase in P-Erk1/2 and suppression of Ki67 staining were additionally reversed with anti-CD86 neutralizing antibody treatment (Figure 4B-4D).

### Polarized myeloid cells regulate early progression of lung cancer

**Figure 5:**
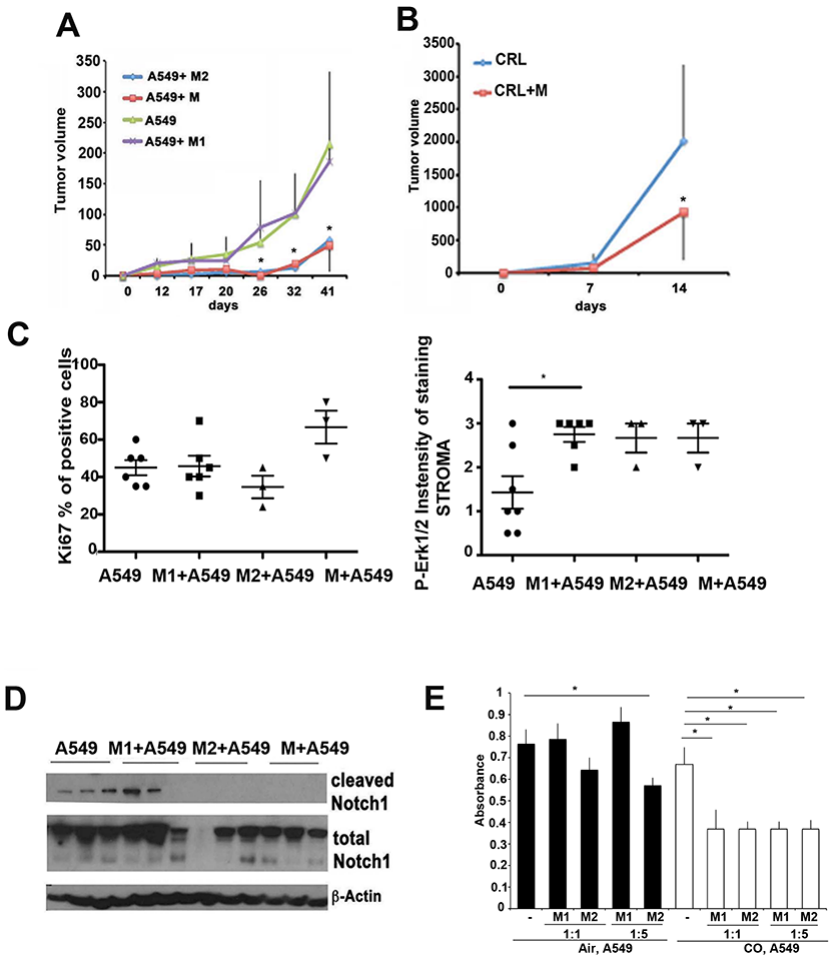
Macrophage polarization influences lung cancer initiation and progression **A–B.** Tumors were established from co-injected A549 lung carcinoma (A, xenografts) or CRL Lewis lung carcinoma (B, synegenic tumors) and bone marrow derived macrophages (BMDM) polarized to M1 or M2 phenotype *in vitro* or non-polarized (M). Growth of tumors was evaluated over the period of 41 days in A549 xenografts and for 2 weeks in CRL xenografts. *p<0.05, A549+wt or M2 macrophages versus A549. **C.** Quantification of immunohistochemistry with antibodies against Ki67 and P-Erk1/2 was performed on established A549 xenografts as in A. **D.** Western blot with antibodies against HO-1, P-Erk1/2, cleaved Notch1 and total Notch1 in A549 xenografts as in A. Data are representative for n=3 per group. **E.** *In vitro* coculture of A549 cells and M1-like and M2-like BMDM for 24 hours in the ratio 1:2 or 1:5 co-treated with CO (250 ppm) or air. *p<0.05, M2 versus non-treated A549 or CO versus CO+M1/M2. Data are representative for n=3 experiments in duplicates.

Balance of macrophage polarization within the tumor microenvironment in Kras mice and A549 xenografts treated with CO led us to investigate whether presence of polarized myeloid cells early in the tumor development may influence cancer growth. We used a standard protocol for polarization of M1-like (CD86^high^/CD197^high^) and M2-like (MMR^high^) macrophages in the presence of INFγ/LPS or IL-4 *in vitro* and showed increased CD86 and MMR expression respectively (data not shown). Non-polarized MCSF-differentiated bone marrow derived macrophages were used as control. Both non-polarized (M) or M2 myeloid cells were treated with macrophage colony stimulating factor (MCSF) for induction of differentiation, however, activation of M2 markers was further increased by treatment with IL-4 for additional 3 days, leading to 20% increase in MMR expression over that induced by MCSF. We injected CRL or A549 cells with or without polarized or non-polarized macrophages subcutaneously into syngeneic C57/BL6 or nude mice, respectively, and followed tumor growth (Figure 5A, 5B). We first confirmed that macrophages (GFP+) remained present in CRL tumors in the tumor microenvironment after 1 and 2 weeks of inoculation and that they maintained their polarization status *in vivo* (Supplementary Figure S4). We observed that co-injection of A549 lung carcinoma cells with non-polarized or M2-polarized (MMR-positive) macrophages slowed down tumor growth unlike when co-injected with M1-like (CD86/CD197-postive) macrophages (Figure 5A). However, the proliferation rate as measured by Ki67 staining did not differ between groups (Figure 5C). These data indicate that tumor growth inhibition in the presence of CO is probably regulated by the polarization phenotype of macrophages as well as that the phenotype of macrophages is different in the presence of CO as compared to that induced by LPS/INFγ. The presence of M2 and non-polarized macrophages blocked cleavage of Notch1, a marker of M1 skewing (Figure 5D, Supplementary Figure S5A–S5B) but did not affect activation of Erk1/2 (Figure 5C). Co-inoculation of M1 macrophages with A549 cells increased P-Erk1/2 expression in tumor stroma (Figure 5C) but did not affect growth of the tumors (Figure 5A). These data suggest that the impact of M1/M2 skewing on tumor growth might be different than the effect of CO treatment. In line with our *in vivo* data, co-cultures show M2-like macrophages blocked A549 proliferation *in vitro* while M1-like macrophages did not affect A549 growth (Figure 5E). CO treatment significantly suppressed growth of A549 cells in the presence of macrophages regardless of their activation or functional status prior treatment (Figure 5E). This indicates that CO dictates macrophage anti-tumor activity regardless of their polarization status.

### HO-1 interacts with Notch1 and negatively correlates with P-Erk1/2 in lung cancer

**Figure 6:**
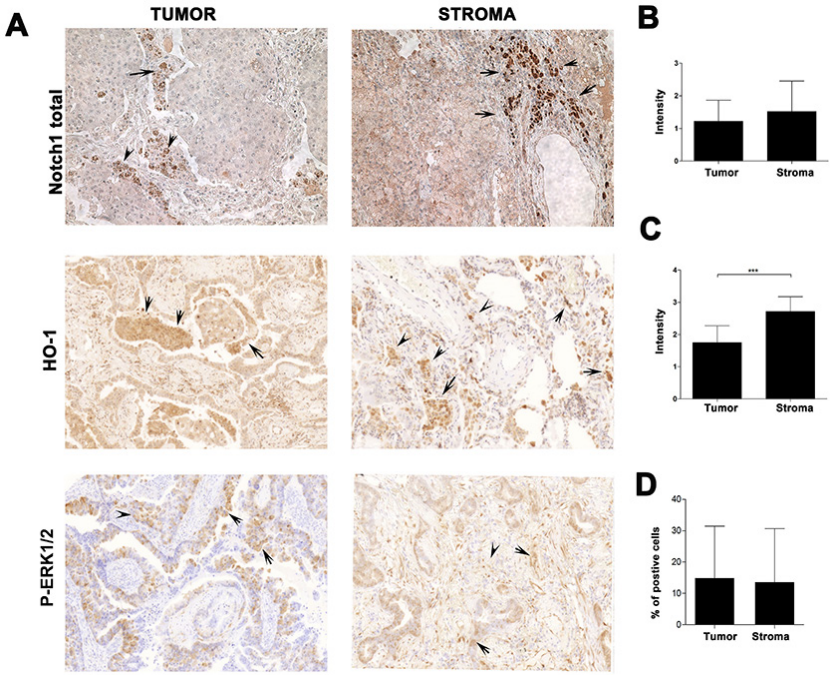
Expression of HO-1, Notch1 and P-Erk1/2 in the stroma of lung cancer patients. Immunohistochemistry analyses of n=30 human patients with dissectible lung carcinoma in the tumor or stroma areas. Total Notch1, HO-1 and P-Erk1/2 stainings are shown in **A.** and quantification is presented in **B–D.** Intensity of staining was evaluated on a scale of 0 as being negative to 4 being the strongest positive. ***p<0.01; stroma versus tumor HO-1 expression. Arrows indicate cells positive for specific markers.

HO-1 and Notch1 are expressed in stroma cells as well as in cancer cells (Supplementary Figure S6). HO-1 is strongly associated with anti-inflammatory, phagocytic and pro-angiogenic macrophage phenotype [22, 23]. HO-1 expression in lung cancer is higher in stroma than in cancer cells (Figure 3A & 3C; Figure 6C). Further, CO blocked HO-1 expression in Kras lung tumor stroma (Figure 3A-3C). To evaluate the expression of HO-1 in primary tumors, we employed human lung carcinoma specimens from 30 patients and performed immunohistochemical analysis of HO-1, Notch1 and P-Erk1/2 in these samples (Figure 6). We found high expression of HO-1 in tumor stroma and a negative correlation between stroma HO-1 and P-Erk1/2 (p=0.01, correlation coefficient by Spearman test, R^2^ = −0.47822), indicating that HO-1 might be a negative regulator of P-Erk1/2 signaling in the tumor microenvironment and/or cancer (Figure 6 and Figure 1, 3).

**Figure 7:**
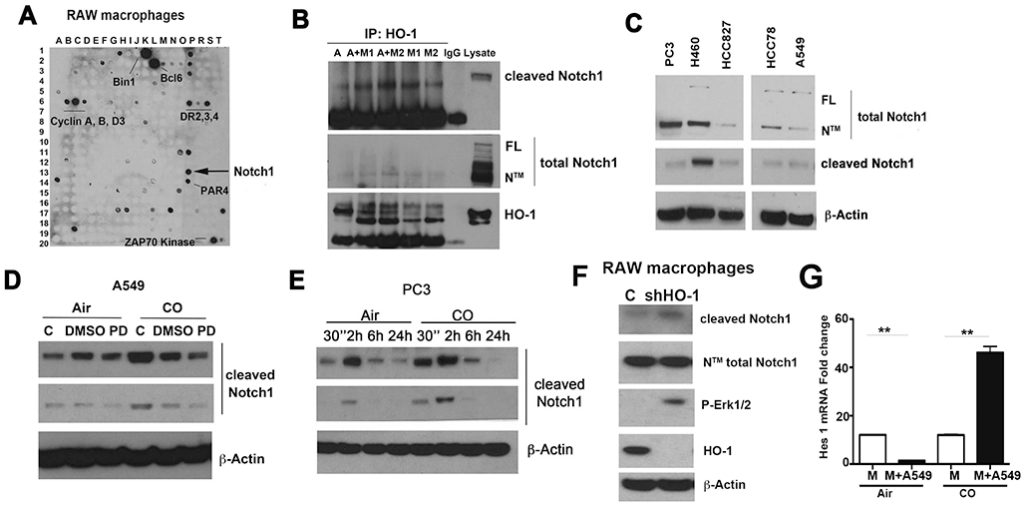
Heme degradation pathway crosstalks with Notch1 signaling **A.** ELISPOT analysis of HO-1 interacting proteins in RAW macrophages. **B.** Immunoprecipitation with antibody against HO-1 in A549 alone or in co-culture with M1 or M2 polarized BMDM. Detection of cleaved and total Notch1 (FL –full length protein, N™-cleaved transmembrane/intracellular region) in the immunoprecipitates was performed by western blot. IgG is a negative control; lysate- A549 cell line. **C.** Expression levels of cleaved and total Notch1 in prostate (PC3) and lung cancer lines (H460, HCC827, HCC78, A549). **D.** Immunoblotting with antibodies against cleaved Notch1 in lysates of A549 cell lines treated with PD98059 (50 mM) for 1h prior CO (250 ppm) treatment for 24 h. **E.** PC3 cells were treated with CO (250 ppm) for 30 min-24h and levels of cleaved Notch1 were measured by western blot. **F.** RAW264.7 macrophages were stably transfected with micro-adapted shRNA against HO-1 (shRNA HO-1) and control vector (C) using retroviral transfer. Stable clones were harvested for western blot analysis. Data are representative for 3 independent measurements. **G.** Real time PCR with primers against Hes1 was performed using mRNA isolated from A549 cells co-incubated with BMDM (wt) in a ratio 1:5. Co-culture was performed for 24h in the presence of CO (250 ppm) or air (untreated control). **p<0.01 wt versus wt+A549.

To further elucidate the role of Notch1 and Erk1/2 signaling and the regulatory role of HO-1 in the tumor microenvironment, we performed an ELISPOT assay in which we identified HO-1 interacting proteins. We found the following proteins to interact with HO-1 in RAW macrophages: Cyclin A, B, D3, DR 2,3,4, Bcl6, BID, Bim, BMX, Bin1, Maspin, MAD2, Notch, Sp1, ZAP70 Kinase (part of TCR), SIVA (ligand for CD4), E-selectin, Syntaxin 6, CAS, ARC and ALK (Figure 7A). Since Notch was identified to be one of the partners of HO-1 and Notch1 cleavage was modulated by CO in Kras and A549 xenograft tumors, we tested the role of HO-1/CO on activation of Notch1. First, we used A549 cells and M1 or M2-like macrophages and their co-cultures’ lysates for immunoprecipitation. We found HO-1 to interact with NTM transmembrane subunit of Notch1 but not with cleaved Notch1 in A549 and M1/M2 macrophages (Figure 7B). Total Notch1 as well as cleaved Notch1 was detected in lung and prostate cancer cells (Figure 7C). To evaluate whether activation of Notch1 is affected by CO *in vitro*, we treated A549 and PC3 cells with CO and showed that cleavage of Notch1 occurs at 30 minutes up to 24 hours after treatment (Figure 7D-7E). Further, since we observed a strong correlation between CO and Erk1/2 phosphorylation, we tested whether MAPK-Erk1/2 is important for CO-induced Notch1 processing. Pretreatement with the Erk1/2 inhibitor, PD98059, blocked CO-induced cleavage of Notch1 (Figure 7D) suggesting a role for MAPK in Notch1 activity.

To further evaluate the role of HO-1 in regulation of P-Erk1/2 and Notch1 signaling, we established RAW macrophages expressing shRNA against HO-1 and showed high expression of P-Erk1/2 and Notch1 cleavage in the absence of HO-1 (Figure 7F). These data further suggest that HO-1 may be a negative regulator of Notch1 signaling and that CO, by blocking HO-1 expression in stroma cells of the tumors (Figure 3A & 3C), releases the negative regulation of P-Erk1/2 and Notch1 signaling. Further, CO induced expression of downstream Notch1-regulated gene Hes1 in the co-culture of A549 and BMDM (Figure 7G).

## DISCUSSION

Our studies suggest that CO blocks tumor growth at least in part via modulation of macrophage phenotype and activity. We showed the importance of a CD86-positive population of myeloid cells in controlling CO anti-tumor responses. Our studies not only provide solid preclinical testing of exogenous CO in models of lung cancer but also indicate that MAPK-Erk1/2→Notch1 signaling dominates in the tumor microenvironment upon treatment with exogenous CO and might be critical for suppression of tumor growth (Supplementary Figure S7). We speculate that CO may target TAMs and macrophages in various polarization and metabolic states (glycolytic M1-like versus M2-like based on oxidative phosphorylation) and dictates their switch towards an anti-tumoral phenotype.

We found that two CO regimens tested at various kinetics may influence cancer growth by changing the tumor microenvironment. Interestingly, low dose of CO at 100 ppm was effective in suppressing tumor xenograft growth and increasing expression of CD86 on infiltrating/residential macrophages. Increased number of CD86-positive cells might serve to activate T and NK cells and initiate an anti-tumoral response. In contrast, higher doses of CO induce infiltration of Gr-1-positive cells into the tumor microenvironment. The effect of CO on myeloid and innate immune cells is likely one of the reasons behind its efficacy as an anti-tumoral gas [8, 19]. Indeed, *in vitro* presence of macrophages in combination with CO treatment allowed for efficient blockage of cancer cell growth as compared to CO alone. This data indicate the necessity of CO for efficient killing of tumor cells by macrophages. Moreover CO alters the phenotype of either M1-like or M2-like macrophages and therefore may restore the balance in the hypoxic tumor microenvironment and induce apoptosis of cancer cells. We showed that efficiency of CO is directly linked to signaling through Erk1/2 and Notch1 pathways in tumor stroma. Inhibition of tumor growth by CO correlated with induction of P-Erk1/2 and cleavage of Notch1. Notch1 signaling has been recently linked to an M2-like phenotype [24], although others have shown that increased Notch1 cleavage is required for macrophage M1 skewing [25]. Further, our data with co-inoculation of M1 that did not suppress tumor growth, unlike CO treatment alone that did suppress tumor growth, suggest that exogenous CO alters macrophage function to anti-tumoral beyond the scope of M1-skewing that can be detected by surface markers. CO treated macrophages may exhibit unique features that are not strictly correspondent to an M1 or M2 phenotype. Further, CO might activate additional pathways in addition to ERK1/2-MAPK and Notch1. Therefore M1-skewing in the absence of exogenous CO may not be sufficient to exhibit anti-tumoral effects. Our data suggest the M1/M2 polarization, CO effects on myeloid cells, and their influence on tumor growth is more complex than is currently recognized in the literature.

Colony Stimulating Factor-1 (CSF-1) signals through Erk1/2→c-myc pathway allowing for proliferation, survival and differentiation of myeloid cells [26]. TAMs lacking c-myc showed delayed maturation and attenuated pro-tumoral functions associated with impaired tissue remodeling, angiogenesis, and melanoma tumor growth in *LysM-Cre:c-myc^fl/fl^*mice with conditional deletion of c-myc specifically in macrophages [27]. We recently showed that HO-1-derived CO and exogenous CO accelerated myeloid cell maturation in part via CSF-1 signaling [2]. In present study, we showed that P-Erk1/2 signaling upon CO treatment in macrophages depends in part on ROS generation. CO is a known inducer of mitochondrial ROS in cancer cells [8] and macrophages [9]. Increased activity of mitochondria in response to CO in otherwise glycolytic TAMs may change their phenotype and lead to suppression of tumorigenesis. We observed different expression of Notch1 and HO-1 in stroma and cancer; it is likely that CO targets similar receptors, namely mitochondrial hemeproteins, however downstream pathways may differ in these two cell types. We have previously reported that CO targets mitochondria and stabilizes Hif1α via mitochondria-derived ROS production in macrophages [9]. We found a similar target of CO in mitochondrial respiratory complexes in prostate cancer cells [8]. However, treatment of cancer cells with CO leads to their ultimate metabolic exhaustion and death, while CO prevented macrophages from apoptosis induced by hypoxia PMID: 17360382 and likely induced their expansion via P-Erk1/2-c-myc pathway. We speculate that the difference in outcomes may be due to different baseline metabolic or activation phenotypes of cancer cells versus macrophages. Therefore, expression of HO-1 and Notch1 in response to CO differs and is likely due to differential activity status of these cells. ROS levels are elevated in cancer cells and further elevation is often linked to apoptosis [8]. ROS are higher in myeloid cells in the tumor microenvironment [28]. Low ROS or hypoxia induces differentiation of myeloid suppressor cells to macrophages and dendritic cells [29]. Futher, myeloid cells are recruited to the tumor based on the hypoxic signal [30]. Importantly, macrophage M2 polarization by hypoxia is dependent on Erk1/2 signaling [31]. Other studies suggest that treatment of mice with long-term hypoxia suppressed A549 tumor growth [32]. Our studies show that M2 macrophages delay tumor growth at the early stages of cancer development. CO exposure may mimic effects of hypoxia in macrophages and block tumor growth dependent on mitochondrial ROS and Erk1/2-regulated macrophage reprograming. Indeed presence of CO efficently promoted macrophage-dependent supression of A549 cancer cell growth *in vitro*. Part of these effects may depend on HO-1 blockade by CO as one of the important anti-tumoral mechanisms. Increased levels of HO-1 in lung cancer was associated with low survival of patients with lung carcinoma and HO-1 was defined as a mediator of ATF-4-dependent anoikis resistance and metastases [33].

We found that P-Erk1/2 is implicated in regulation of Notch1 activity upon CO treatment. Notch1 is not only important in macrophage differentiation but also in their polarization towards an M1-like phenotype [34]. Indeed, Notch1 cleavage is triggered in response to toll like receptor ligands (e.g. LPS) that induce a proinflammatory macrophage phenotype [25]. However, LPS/INFγ skewed macrophages had limited effect on lung cancer growth and progression when injected at the time of tumor initiation. One of the explanations for this observation may be that M1-like macrophages have higher plasticity in comparison to M2-like or non-polarized macrophages [35].

HO-1 is a well-established protein involved in conferring a M2-like phenotype in macrophages [36, 37], however functional studies in tumor models *in vivo* are limited. We showed that HO-1 in macrophages dictates their differentiation and polarization to M2 or M1-like phenotype [2, 5]. CO differentially modulates HO-1 expression during inflammation, inducing its expression in the liver while blocking it in the lung [38]. We find that CO blocks HO-1 expression in the tumor microenvironment of lung cancer xenografts as well as in Kras tumors. Further, analyses of human lung cancers from 30 patients showed that HO-1 is negatively correlated with P-Erk1/2 in the stroma tissues indicating the possible implication of HO-1 in the effects of CO on P-Erk1/2 and Notch1 signaling. It is possible that CO negatively regulates HO-1 expression to prevent further activity in the tumor microenvironment or by switching the phenotype of cells from M2 to M1 so fewer HO-1 expressing cells are present in the tumor. We speculate that HO-1 may interact with Notch1 to prevent its cleavage until CO is present. HO-1 is associated with metastases and poor overall survival of patients with lung carcinoma [33]. CO in turn inhibits HO-1 expression and therefore releases the inhibitory effects of HO-1 on the Notch1 pathway.

We have shown that CO negatively regulates HO-1 in macrophages. CO is involved in the regulation of hypoxia-induced gene expression. Morita et al. reported that CO inhibited induction of HO-1 gene transcription during hypoxia via a negative feedback mechanism [39]. Further, the presence of CO suppressed hypoxia-induced HO-1 gene expression by ~54% with no effect on the induction of HO-1 steady-state mRNA levels in response to exogenous NO [40]. Thus, part of the mechanism of CO in macrophages might involve regulation of HIF-1α-dependent HO-1 expression.

Our studies suggest that CO induces early cleavage of Notch1 and decreases the amount of total Notch1. Notch1 is strongly associated with tumor progression in patients with leukemia or T-cells lymphoma [41, 42] and crosstalks with c-myc pathway [43]. Notch1 dictates mitochondrial metabolism reprograming towards proinflammatory M1 macrophages [44]. We speculate that the mechanism of CO-induced Notch1 activation is likely through increased mitochondrial ROS in macrophages [9] or cancer cells [8]. Notch1 signaling is induced [45] and mediates protection against cell death in response to oxidative stress [46].

Since inflammation is a hallmark of cancer, modulation of innate cells as well as their interaction with cancer cells may prove to be a novel strategy to treat cancer. CO is a potential player in regulation of an immune environment in the tumor thus low non-toxic doses may be used as a potential therapy for advanced cancer.

## MATERIALS AND METHODS

### Reagents

Cell culture media (DMEM high glucose, RPMI 1640), sterile PBS, Trypsin-EDTA and antibiotics (Antibiotic-antimycotic; Penicillin/Streptomycin) were purchased from Life Technology/Invitrogen (Carlsbad, CA, US). Fetal bovine serum (FBS) was from Atlanta Biologicals (Flowery Branch, GA). Primary and secondary antibodies used for immunohistochemistry, immunoblotting and flow cytometry and are as follows: anti-HO-1 (Abcam, Cambridge, MA), anti-β-Actin (Sigma-Aldrich, MO), anti-Notch1 and anti-cleaved Notch1 (Cell Signaling, Beverly, MA; Novus Biologicals; Proteintech), anti-c-myc, anti-P-MAPK-Erk1/2, anti-P-Akt, anti-P-Retinoblastoma (Rb) and anti-P-Histone H3 (Cell Signaling, Beverly, MA), anti-mTFA, anti-P-Elk1/2 and anti-Elk1 (Santa Cruz Biotechnology, Dallas, Texas), anti-cyclin D1 (Calbiochem, Millipore, USA), anti-CD86 and anti-MMR (Biolegend, San Diego, CA). SuperSignal West Femto/Pico Substrate reagents for Western-blot were purchased from Fisher Scientific. Recombinant M-CSF, IL-4 and IFNγ were purchased from Peprotech. LPS (*E.coli* 0127:B8) was from Sigma. Anti-CD86 neutralizing antibodies were purchased from Biolegend. Pegylated catalase (3000 U/mL) and SOD (10 U/mL) were from Sigma and were used 1 hour before CO treatment as previously described [8].

### Animals

All experimental procedures were performed in accordance with relevant guidelines and regulations. All experiments were approved by the Institutional Animal Committee, IACUC at BIDMC.

#### Kras model

FVB/N-Tg(teto-Kras2)12Hev/J lung adenocarcinoma mice (Kras) were used to investigate CO effect on tumor development as previously described [8]. Five weeks old mice were given doxycycline food for 13 weeks followed by treatment for an additional five weeks with CO (250 ppm) daily for 1h. C57BL/6 mice (Jackson Laboratory, Bar Harbor, ME) were used as bone marrow donors.

#### Xenograft models

Nude mice (Taconic, Germantown, NY) or C57Bl/6 mice were injected subcutaneously with 1×10^6^ A549 lung adenocarcinoma cells or CRL cells (mouse Lewis lung carcinoma) with or without BMDM (1:2 ratio), respectively and tumors were established for 2 weeks-1 month until the volume was 100 mm^3^. CO was applied at 100 ppm or 250 ppm daily or twice per week or only once per week for 1h. Mice were placed in the plexiglass CO chamber with available food and water ad libitum and remained in the chamber for the duration of the exposure for 1h. CO was pre-mixed with air before entering the chamber to achieve 250 ppm. This concentration lead to increased carboxyhemoglobin levels of 10-15 % in the blood after exposure for 1h. Neutralizing antibody (100 mg anti-CD86 per 25g mouse) was given by intraperitoneal injection to mice with established xenografts just prior to CO exposure at 100 ppm for 1h. Mice were treated with antibody and CO (1h, 100 ppm) twice per week for 2 weeks.

### Human specimens

Frozen, zinc and formalin-fixed paraffin embedded tissues were used as previously described [47]. The thirty human formalin-fixed paraffin embedded (FFPE) lung adenocarcinoma samples in a form of the tissue microarray (TMA) were obtained from the tissue bank of the Department of Pathology at the National Koranyi Institute of TB and Pulmonology, Budapest, Hungary (ETT-TUKEB no.:2521-0 2010-1018EKU (153 PI 010). Informed consent was obtained from all patients, and all procedures were approved by the Institutional Ethics Committee (2521-0 2010-1018EKU).

### Cell culture

Human A549, HCC78, HCC827 and PC3 cell lines as well as mouse RAW264.7 macrophage cell lines were purchased from ATCC and cultured in RPMI media supplemented with 10% FBS and 100 mg/ml penicillin/streptomycin solution. Bone-marrow derived macrophages (BMDM) were differentiated in RPMI 1640 media supplemented with 20 ng/ml M-CSF, 15% FBS and antibiotic-antimycotic solution as previously described [47]. In other experiments, BMDM were further polarized using M1 or M2 skewing protocols: IFNγ (10 ng/ml ) + LPS (100 ng/ml) or IL-4 (10 ng/ml) for 3 days, respectively. Cells were washed once with PBS and used for subsequent experiments.

RAW264.7 macrophages were stably transfected with micro-adapted shRNA against HO-1 and control vectors as previously described [10, 48].

### Immunohistochemistry

Zinc and FFPE samples were deparaffinized followed by the antigen retrieval procedure. Human FFPE samples in TMA as well as mice samples for staining of HO-1 and P-Erk1/2 were retrieved by high pressure cooking (HPC; 2100-Retriever, EMS, Hatfield, PA, CatNo. 62706) in citrate buffer (pH 6.0) for 1 hour; Zinc fixed xenograft samples were retrieved in DMSO for 3 minutes for CD86 and Gr-1 staining. Acetone was used after fixation of sections to detect NK antigen. After washing with 1x PBS, slides were blocked with 7% horse serum (Normal Horse Serum, VectorLabs, Peterborough, UK). Primary antibodies were applied overnight at 4¼C. The following day, slides were washed once in 1x PBS, blocked with H_2_O_2_, and then washed three times in 1xPBS for five minutes per wash. Biotin-labeled secondary antibodies were applied for an hour at room temperature. VECTASTAIN Elite ABC System was used to enhance the signals (VectorLabs). DAB substrate (VectorLabs) was used to develop the reactions, followed by slide dehydration, mounting, and analysis by light microscopy. Microphotographs were taken under 100-200x magnifications in the light microscope (Nikon). Immunostainings were quantified by evaluating percent of positive staining or number of cells or intensity of staining per field of view using Soft View software. Details are provided in Figure legends.

### Flow cytometry

The following antibodies were applied for staining: CD86-APC, CD197-PE, MMR-PE and CD68-APC macrophage markers (Biolegend, San Diego, CA). All antibodies were incubated with cells for half an hour at room temperature at 1:100 dilution. BD FACS Calibur (BD Biosciences, San Jose, CA) was used for acquisition of the staining and CellQuest for subsequent analysis.

### BrdU

BrdU proliferation assay was purchased from Roche Applied Science (Mannheim, Germany;) and applied according to manufacturer's protocol.

### Immunoblotting

Proteins were harvested in RIPA buffer (25mM Tris-HCl, 150mM NaCl, 1%NP-40, 1% sodium deoxycholate, 0.1% SDS, 200mM PMSF, 100mM NaF, Complete Mini Protease Inhibitor Cocktail Tablets, Roche Applied Science). After sonication, lysates were centrifuged at 14,500 rpm at 4°C for 20 minutes. 20-30 mg proteins were used for electrophoresis using pre-casted gels (NuPAGE, Life Technologies) in MES-SDS running buffer (Life Technologies). Transfer of resolved proteins was performed in wet transfer chambers using 20% methanol-Tris-glycine buffer for 1.5 h at 80V. Membranes were blocked in 5% non-fat milk for 1 hour. Primary antibodies were applied for overnight at 4°C. After washing in 1x TBS blots were incubated HRP-labeled secondary antibodies (Cell Signaling) for an hour at RT. Chemiluminescent substrates (Fisher Scientific) and X-ray films were applied for detection. Quantification was performed using Image J64 software (NIH).

### RNA isolation and real time PCR

Total RNA was isolated using Qiagen RNasy Mini Kit (Qiagen) following manufacturer's protocol. The RNA concentration was measured by NanoDrop-1000 (Fisher Scientific). BioRad iScript cDNA kit was used to prepare cDNA from 1-2 mg of total RNA. The reverse transcription reaction steps were as follows: 5 minutes at 25°C, then 30 minutes at 42°C, 5 minutes 85°C and hold at 4°C.

Sybr Green PCR Master mix from Life Technology was used for RT-PCR reactions. The following primers were used: Hes1 F 5’TGGAAATGACAGTGAAGCACCT 3’, R 5’GTTCATGCACTCGCTGAAC 3’; β-Actin F 5’ CCACAGGATTCCATACCCAAGA3’, R 5’TAGACTTCGAGCAGGAGATGG 3’.

### Statistical analysis

Statistical analysis was performed using GraphPad (GraphPad Prism version 5c, GraphPad Software, La Jolla California USA). T-test and ANOVA were used to compare the groups and *p*<0.05 was considered as significant. The correlation was calculated by Spearman's rank correlation and *p*<0.05 was defined as significant difference between the studied variables.

## SUPPLEMENTARY FIGURES


