# Supplementary material for: Alterations of tumor microenvironment by carbon monoxide impedes lung cancer growth
Source: Oncotarget. 2016 Mar 15;7(17):23919–32. doi: 10.18632/oncotarget.8081 (PMC5029674; doi:10.18632/oncotarget.8081)
Supplement: Supplementary file 1 [file oncotarget-07-23919-s001.pdf]

SUPPLEMENTARY FIGURES

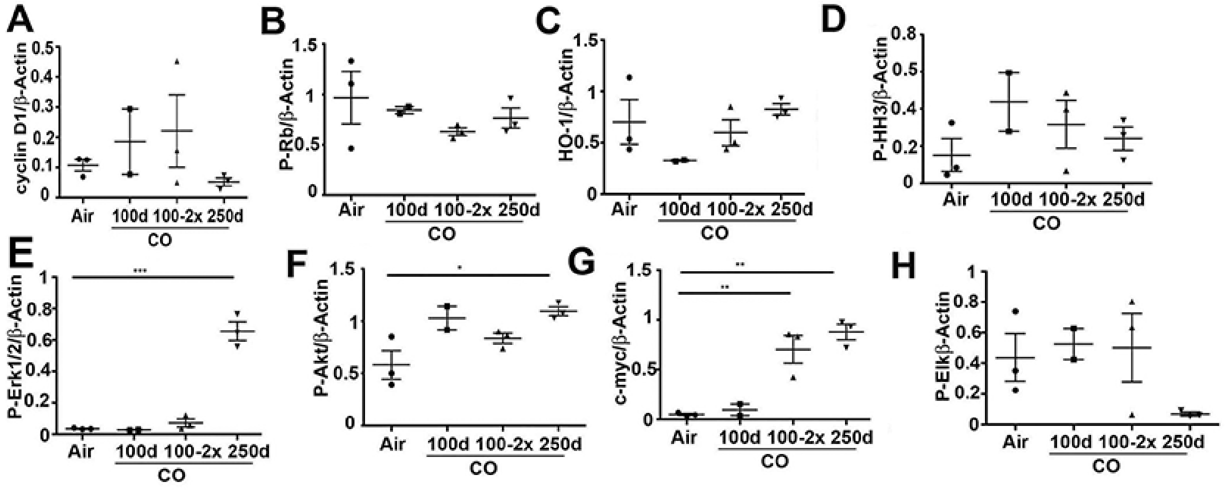

Supplementary Figure S1: A–H. Quantification of immunoblots in Figure 1C was performed using Image J software. p<0.01 ** and p<0.05 *, CO versus air control.

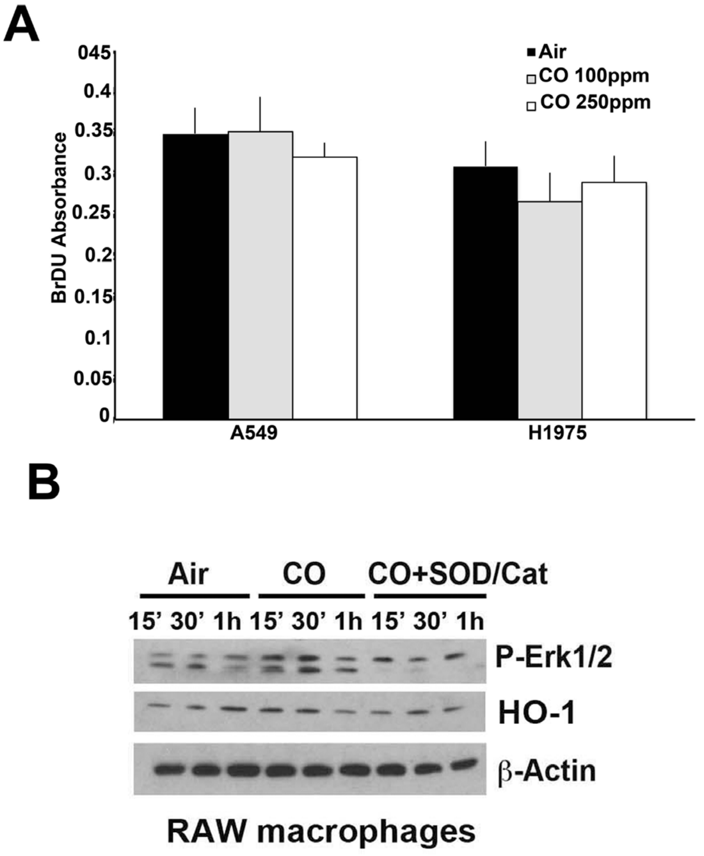

Supplementary Figure S2: A. BrdU incorporation assay was performed in A549 and H1975 lung cancer cell lines treated with CO at 100 ppm and 250 ppm for 24 hours. n=4. Non-statistical differences were noted between CO and air treated samples *in vitro*. **B.** RAW264.7 macrophages were treated with superoxide dismutase-polyethylene glycol and catalase-polyethylene glycol 1h prior CO (250 ppm) exposure for 15 min to 1h. Levels of P-Erk1/2 and HO-1 were measured by immunoblot. Data are representative of 3 independent experiments.

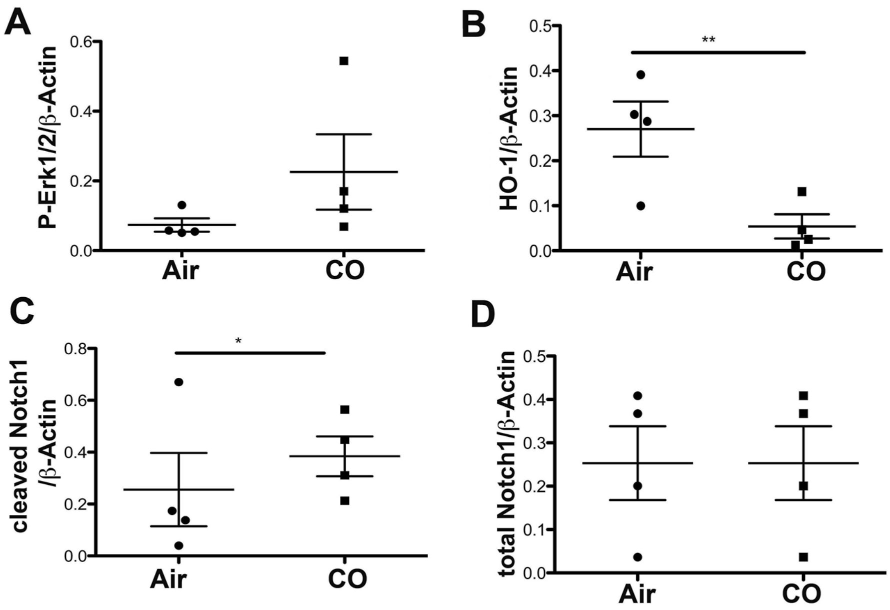

Supplementary Figure S3: A-D. Quantification of immunoblots in Figure 3E was performed using Image J software. p<0.01 ** and p<0.05 *, CO versus air control. Note: One outlier from air treated mice was removed from statistical analysis for evaluation of the levels of expression of Notch1 cleaved (p=0.04) and HO-1 (p=0.0012).

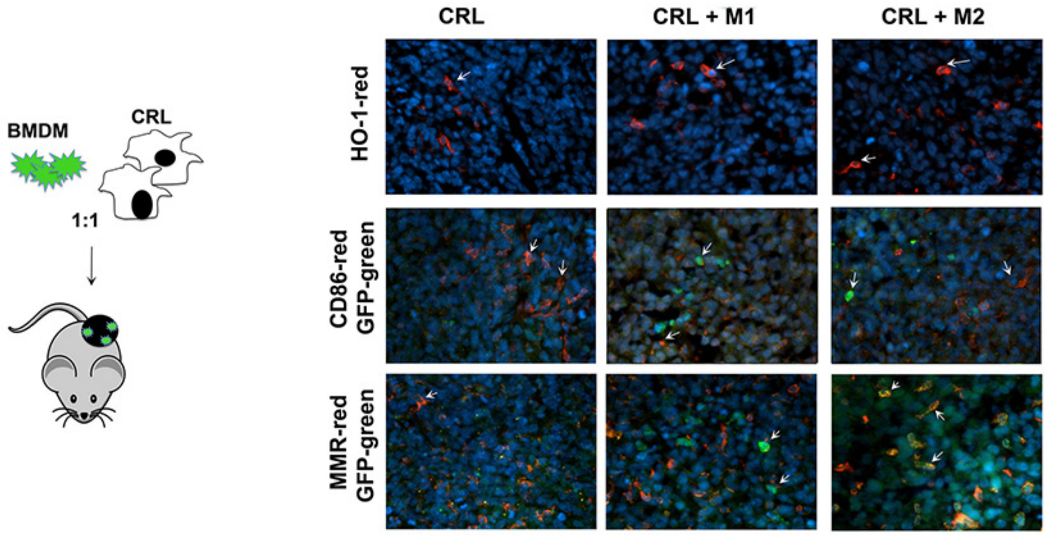

Supplementary Figure S4: CRL lung carcinoma cells with polarized or unpolarized macrophages from GFP+ transgenic mice were injected into C57BL/6 mice. Immunohistochemical detection of M1 marker, CD86 (red) or M2 marker, MMR (red) of injected macrophages (green GFP) in CRL xenografts. Overlapping of green and red staining showed that M1 and M2 macrophages sustained their polarity in xenograft tumors 1 week post-injection. We also detected HO-1 expression in these cells.

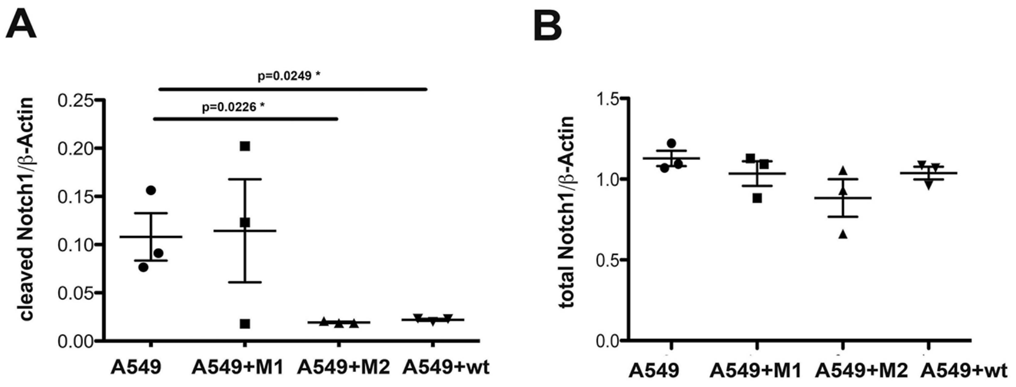

Supplementary Figure S5: A–B. Quantification of immunoblots from Figure 5A was performed using Image J software. *p=0.0249 A549 versus wt+A549, *p=0.0226, A549 versus A549+M2 by T-test.No statistically significant differences were noted between the samples by one-way ANOVA test (p=0.085) and Tukey's multiple comparison test for A or B.

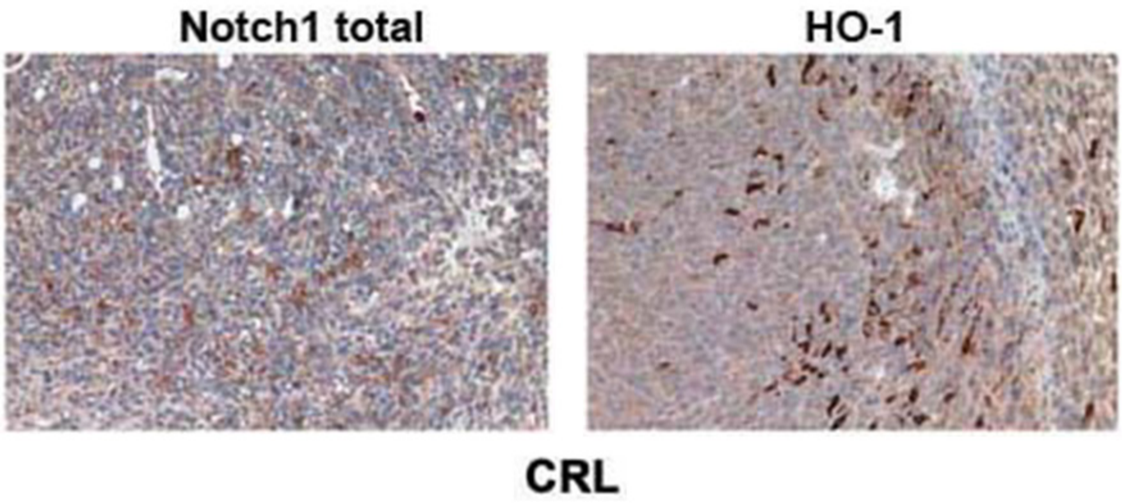

Supplementary Figure S6: CRL xenografts were stained with antibody against Notch1 total or HO-1 and representative pictures are shown.

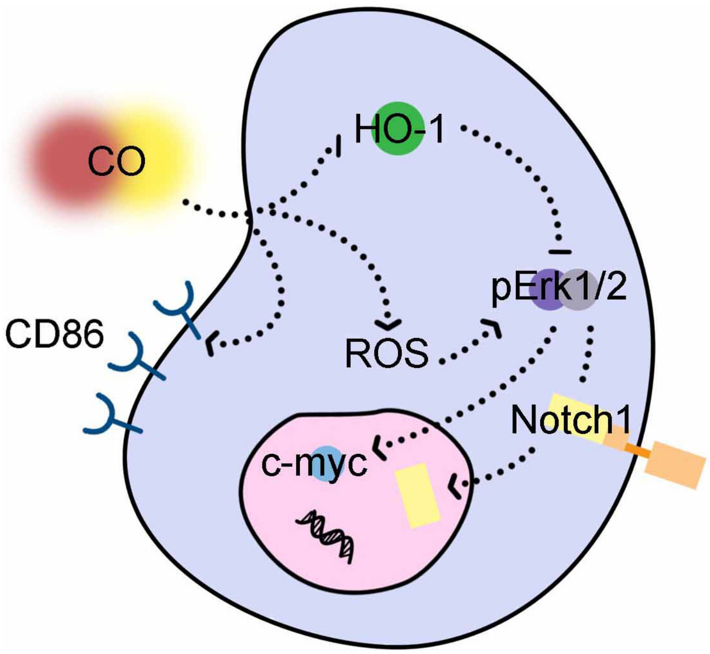

Supplementary Figure S7: Scheme cartoon on the role of CO in myeloid cell in tumor microenvironment. CO activates MAPK-Erk1/2→Notch1 signaling via ROS-dependent mechanism. Furthermore, blockade of HO-1 expression by CO further amplifies P-Erk1/2 signaling. CO warrants a balance in tumor microenvironment and suppression of tumor growth by regulation of CD86-positive myeloid cells.
